# Wearable Arduino-Based Electronic Interactive Tattoo: A New Type of High-Tech Humanized Emotional Expression for Electronic Skin

**DOI:** 10.3390/s25072153

**Published:** 2025-03-28

**Authors:** Chuanwen Luo, Yan Zhang, Juan Zhang, Linyuan Hui, Ruisi Qi, Yuxiang Han, Xiang Sun, Yifan Li, Yufei Wei, Yiwen Zhang, Haoying Sun, Ning Li, Bo Zhang

**Affiliations:** 1 Department of Architecture, School of Architecture and Art, North China University of China, Jinyuanzhuang Road 5, Shijingshan District, Beijing 100144, China; witchwen@ncut.edu.cn (C.L.); zy2021@mail.ncut.edu.cn (Y.Z.); juanzrf@ncut.edu.cn (J.Z.); huilinyuan@mail.ncut.edu.cn (L.H.); qiruisi-77@mail.ncut.edu.cn (R.Q.); hanyuxiang@mail.ncut.edu.cn (Y.H.); sunxiang23@mail.ncut.edu.cn (X.S.); liyifan@mail.ncut.edu.cn (Y.L.); weiyufei_gessica@mail.ncut.edu.cn (Y.W.); zhangyiwen88@mail.ncut.edu.cn (Y.Z.); sunhaoying@mail.ncut.edu.cn (H.S.); 2 Beijing Historical Building Protection Engineering Technology Research Center, Beijing University of Technology, Beijing 100124, China

**Keywords:** electronic interactive tattoo, e-skin, Arduino Nano, sensor integration

## Abstract

Skin is the largest organ of the human body and holds the functions of sensing, protecting, and regulating. Since ancient times, people have decorated their skin by painting themselves, cutting, and using accessories to express their personality and aesthetic consciousness as a kind of artistic expression, one that shows the development and change of aesthetic consciousness. However, there are concerns regarding the inconvenience, high time cost, and negative body perception with traditional tattoos. In addition, the trend of skin decoration has gradually withdrawn due to a lack of intelligent interaction. In response to these problems, we proposed a wearable electronic skin tattoo that offers a novel means of communication and emotional expression for individuals with communication impairments, WABEIT. The tattoo uses skin-friendly PDMS as the base material, combines multi-mode sensing components such as silver wire circuit, a programmable Surface-Mounted Device (SMD), a thin-film-pressure sensor, and a heart rate sensor, and combines the embedded development board Arduino Nano for intelligent interaction, forming a wearable electronic interactive tattoo capable of sensing the environment, human–computer interaction, and the changeable performance of intelligent perception. The sensor is also equipped with a mobile power supply to support portability. The advantages of WABEIT are as follows: first, it avoids the pain, allergy, and long production process of traditional tattoos. Second, the patterns can adapt to different needs and generate feedback for users, which can effectively express personal emotions. Thirdly, the facility of removal reduces social discrimination and occupational constraints, which is especially suitable for East Asia. Experimental results indicate that the device exhibits a high sensitivity in signal response, a wide variety of pattern changes, and reliable interactive capabilities. The study demonstrates that the proposed design philosophy and implementation strategy can be generalized to the interactive design of other wearable devices, thereby providing novel insights and methodologies for human–computer interaction, electronic devices, and sensor applications.

## 1. Introduction

### 1.1. History of Skin Decoration

Human skin does not own the functions of communication, foraging, or courtship. As the largest organ of the human body, skin possesses characteristics such as extensibility, self-healing ability, and high mechanical toughness [1,2,3,4], which enables us to perceive different shapes and textures, changes in temperature, and varying levels of tactile sensations around the environment. Throughout history, people have been constantly attempting to modify the thickness of the skin to some extent and add decorations and patterns to enhance its appearance in order to achieve diverse information transmission functions related to emotions, culture, and beliefs. Among these, the most prominent performance is tattooing art.

Primitive humans have started to create fixed tattoos with sharp stones, knives, thorns, needles, and other tools to prick different patterns in different parts of the body to form a permanent decoration with green grass juice or coloring matters. Archaeologists have found tattoos on human remains in Alaska, Mongolia, Greenland, Egypt, China, Sudan, Russia, and the Philippines dating back to 2100 BC [5]. One role of primitive human tattoos is to beautify the body; the second is to frighten the enemy, embodying the spirit of primitive people who were not afraid of suffering and dedication to the group; therefore, the tattoo has undoubtedly become a noble and beautiful symbol.

Ancient Egyptian tattoos were mainly found on women’s skin, which might be related to fertility, health, or religious rituals, and were even considered as a medical treatment [6]. The Samoans used turtle shells and wild boar teeth for tattoos to commemorate events such as maturity, honor, family, and courage, which were symbols of personal identity and social status, representing great endurance and dedication to culture [7]. In Ancient China, tattoos were regarded as barbaric; folk heroes and bandits had tattoos, and convicted criminals would also punish with facial tattooing, known as “stigma”, to warn other members of society of their danger and unreliability [8]. Japanese tattooing art originated as a means of camouflage when fishing in water. In the 18th century, Japan followed China’s example and stipulated those tattoos be imposed as punishment for criminals. The Meiji government passed a law in 1870 prohibiting tattoos, considering them a rude and barbaric behavior [9]. Tattoo is a form of body art expression with a long history, which has rich spiritual connotation and aesthetic style, as well as its unique historical inheritance and cultural attributes. However, the methods and materials used to form tattoos can cause irreversible damage to the skin and increase the risk of disease; additionally, employment restrictions and social discrimination exist in some areas. The tattooing patterns have low variability and lack real-time communication and interaction capabilities. These defects make tattoos gradually a problem and difficult to develop.

### 1.2. Literature Review

Owing to the progress of science and technology, people have gradually realized that the skin’s protection of the body’s organs is irreplaceable. People have begun to explore electronic skin, a material that emulates the functions of human skin by sensing environmental changes and converting them into electronic signals for processing [10]. Current research on e-skin mainly falls into two categories as the subheadings show.

#### 1.2.1. Perception Enhancement and Functional Diversity

(1) Enhancement of sensing capabilities

**Figure 1:**
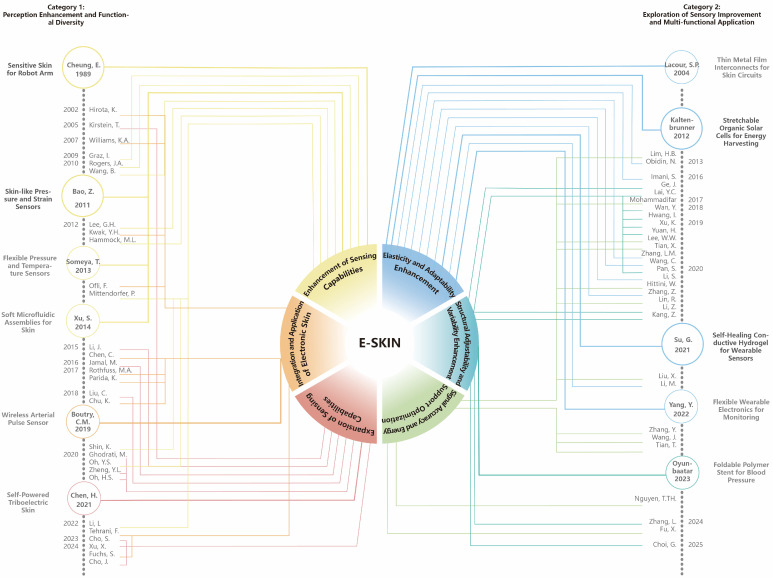
Development of e-skin. Mainly in two categories [11,12,13,14,15,16,17,18,19,20,21,22,23,24,25,26,27,28,29,30,31,32,33,34,35,36,37,38,39,40,41,42,43,44,45,46,47,48,49,50,51,52,53,54,55,56,57,58,59,60,61,62,63,64,65,66,67,68,69,70,71,72,73,74,75,76,77,78,79,80,81,82,83,84,85].

A significant research branch within the field of e-skin is the development of advanced sensing capabilities that emulate human skin’s multifaceted sensory functions. By integrating multiple types of sensors, e-skin is capable of real-time monitoring of various environmental changes, including thermal sensitivity maps for precise temperature perception, the exploration of tactile for enhanced touch sensitivity, improving the spatial discrimination, and other environmental physiological parameters, thereby significantly enhancing its perceptual capabilities (Figure 1) [11].

General Electric (GE) developed the sensitive skin of the robot, which integrates discrete infrared sensors with a sensing range of 3–10 inches to enhance the robot’s environmental perception ability, thus improving its adaptability in uncertain environments [12]. Professor Bao et al. introduced a novel e-skin to diagnosing psychological disorders, such as anxiety and depression, by monitoring cortisol levels in sweat and assessing stress states [13,14]. Graz et al. and Tian et al. achieved an effective distinction between temperature and pressure inputs by integrating piezoelectric and thermoelectric materials [15,16]. The Someya’s team has significantly improved the perception and flexibility of the electronic skin with flexible pressure and temperature sensors designed with field effect transistors (OFETs), achieving up to 230% stretchability without compromising sensor performance [17,18]. Rogers et al. and Xu et al. simulated the tactile perception of human skin by developing stretchable capacitive, piezoresistive, and piezoelectric sensor arrays and used multilayer nanomembrane transfer technology to create highly integrated, flexible electronics to enhance haptic feedback [19,20]. Hammock et al. developed electronic skins that integrate optoelectronic devices such as Light-Emitting Diodes (LEDs), achieving wireless power via resonant inductive energy transfer. These devices can stretch up to 30% and twist by 75%, with electrocardiogram (ECG) signal-to-noise ratios exceeding 50 dB, enhancing their accuracy and sensitivity in biomedical devices and robotic arms [21,22]. Regarding hardness perception, Li et al. developed a dual-sensor system capable of distinguishing materials with current output ranging from 50μA for soft materials to over 200μA of varying hardness, thereby improving the feedback precision of robots and biomedical devices in object operation [23]. As well, Shin et al. proposed a highly sensitive tactile sensor, capable of detecting 3 mm depth changes with 1 mm resolution, that significantly improves the feedback capabilities of robotic arms in complex operations [24]. In addition, the application of electronic skin in soft robots has also made important progress. The ETH Zurich team has provided soft robots with tactile ability similar to human skin, achieving 1 mm resolution in 3D surface detection and improving its operation accuracy in complex environments [25]. Oh et al. developed the battery-free, wireless flexible sensor that enables multi-point pressure and temperature monitoring, thus the application of environmental sensor technology [26].

These advancements aim to enhance the overall perceptual capabilities of electronic skin, making it more responsive and adaptable to various environmental stimuli.

(2) Expansion of sensing capabilities

Not only simulating the functions of human skin, e-skin also expands to new functions that human skin does not have, significantly enhancing e-skin perception. Xu and Yan studied the ultra-sensitive temperature-acoustic photo responsive bionic skin sensor and applied it to the perception and transmission of underwater information [27]. Ghodrati et al. developed CNT-based transistors for specific gas detection, achieving a detection limit as low as 50 ppb for gases like NO_2_ and NH_3_ and significantly improving the selectivity of the sensor and the lower detection limits [28]. Jamal et al. developed a non-enzyme electrochemical sensor using palladium nanoparticles (PdNPs), which significantly improved the equipment stability and self-energy capacity of glucose detection, achieving a sensitivity of 530 μA mM^−1^ cm^−2^ at 20 °C and a detection limit of 5 μM (S/N = 3) [29]. Chen et al. realized the self-energy function of the electronic skin by dispersing the strain and vibration sensors in the sensor skin at different depths. They also were able to collect complex environmental information [30,31]. The MIT and Harvard teams focused on the design of high-density multi-sensor integrated systems and developed multimodal sensor arrays, as well as optimizing data processing and wireless communication protocols, thus driving the development of intelligent data analysis and highly integrated electronic skin systems [32,33]. The KAIST team has developed smart wearable devices that integrate multi-functional sensors such as temperature, humidity, and pressure, further expanding the application of electronic skin in biomedicine [34,35]. In addition, IMEC and several international teams developed scalable electronic skin sensors using 3D printing and inkjet printing, achieving a pressure sensitivity of 0.601 KPa^−1^ and enhancing multi-functionality for wearable devices and flexible electronics [36,37].

These research studies have significantly improved the perception ability, functional diversity, and application range of e-skin, laying a solid foundation for the wide application of e-skin in smart, wearable biomedical and environmental monitoring fields.

(3) Integration and Application of Electronic Skin

Electronic skin (e-skin) technology has made remarkable progress by enabling multiple new functions through innovative materials and designs. Boutry et al. developed a degradable wireless sensor that can be wrapped around blood vessels to continuously monitor blood flow after surgery in 2019, providing a new solution for medical monitoring [38]. Chu et al. developed conductive circuits using liquid metal microcapsules (2–10 µm), enabling electronic skin to self-heal after mechanical damage, enhancing durability. In perovskite solar cells, the passivation film with LMCs retained 99% of the initial power conversion efficiency (PCE = 15.07%) after cutting [39]. Williams et al. improved the durability and energy independence of electronic skin by developing self-healing materials with a conductivity of 10^−3^ S cm^−1^ and the ability to repair microcracks via thermal treatment [40]. Parida et al. developed a transparent, stretchable, self-healing ionic skin triboelectric nanogenerator that provides e-skin with the ability to self-power and touch applications [41]. Additionally, multiple international research teams, such as UCLA and Tsinghua University, have developed non-invasive sensors that combine microneedle technology and biocompatible materials for the detection of human sweat and other biological signals, driving the application of electronic skin in personalized health management and chronic disease monitoring [42,43,44]. Fuchs et al. proposed a glucose-responsive catheter for automated, electronic intervention free insulin delivery [45]. Rothfuss et al. developed an implantable stent for energy harvesting for percutaneous wireless monitoring of peripheral artery disease, demonstrating the application prospect of electronic skin in remote health monitoring [46]. Chen et al. and Ofli et al. at UC Berkeley released the UTD-MHAD dataset for human behavior recognition [47,48], and the University of Tokyo improved the sensor’s recognition accuracy, achieving 100% accuracy in distinguishing plastic from metal cans and 80% accuracy in recognizing three internal states (empty, half, and full), and system adaptability, which promoted the application of electronic skin in multimodal sensing and intelligent systems [49].

E-skin technology has made remarkable progress in perception enhancement, function expansion, and integrated application. By integrating a variety of sensors and advanced materials, e-skin not only mimics the tactile, temperature, and pressure sensing capabilities of human skin but also extends new functions such as magnetic sensing, self-healing, and self-powered. These research studies not only improve the perception ability and functional diversity of electronic skin but also promote its wide application in smart wearable, robotics, and human–computer interaction.

#### 1.2.2. Exploration of Sensory Improvement and Multi-Functional Application

Another major category of research focuses on the bionics and performance optimization of flexible materials, which typically encompass electrode materials, active materials, and flexible substrates.

(1) Elasticity and adaptability enhancement

A prominent area of research is dedicated to the bionics and performance optimization of flexible materials, including electrode materials, active materials, and flexible substrates. Wang et al. developed ultra-thin silicon device using silk substrate, achieving 10^−3^ S/cm conductivity and 200% strain tolerance to enhance biodegradability in e-skin [50]. Su et al. designed self-healing materials and elastic electrodes with 2590% stretchability and 3.35 S/m conductivity that significantly improve the biocompatibility and environmental adaptability of flexible devices for deep integration with biological systems [51]. Researchers at Harvard University developed self-healing nanocomposites for long-term wearable health monitoring sensors, achieving 95% healing efficiency, 2000% stretchability, 347.3 N m^−1^ adhesion, and a gauge factor of 20 under 800–1400% strain. This innovation enhances sensor durability, ensuring reliable performance after 500 cycles and in extreme temperatures from −20 °C to 60 °C, reducing maintenance and replacement needs [52,53,54]. These research studies also encompassed the development and design of biocompatible materials, including hydrogels and functional polymers, as well as the fabrication of flexible sensor structures that are compatible with biological tissues. This significantly improved the compatibility between the sensors and biological tissues, thereby ensuring long-term and stable monitoring of biological signals with an accuracy of 95% over 500 h of continuous use [55,56]. The UCLA team successfully addressed the issues of immune response and mechanical stability by optimizing the biocompatibility of the flexible electronic skin, ensuring the high integration of the sensors with biological tissues and long-term stability [57,58]. Lacour et al. improved the mechanical compliance and durability of electronic skin by utilizing flexible silicon films and stretchable interconnection, achieving 25 nm gold films on elastomeric substrates with 8.4 μm wavelength and 1.2 μm amplitude surface waves and reproducible resistance changes under 15% strain cycling [59]. Meanwhile, Kaltenbrunner et al. substantially enhanced the self-powered capability of electronic skin through the development of stretchable organic photovoltaics (OPVs) on plastic foil substrates less than 2 μm thick, achieving power conversion efficiency equal to glass-based counterparts, and the integration of over ten times thinner, lighter, and more flexible solar cells, making it suitable for wearable devices and flexible electronics applications [60].

(2) Structural adjustability and variability enhancement

From the perspective of structural flexibility [61,62], researchers have designed a new mechanical structure [63,64], aiming to transform traditional materials into deformable materials [65,66,67], so as to improve the deformability and adaptability of electronic skin [68,69,70]. The metal-coated elastomer fiber interlocking structure designed by the Pan et al. significantly improved sensitivity 14.3 times compared to physical stacking, enabling highly sensitive sensing functions for various forces [71]. In addition, the collapsible polymer stent proposed by Oyunbaatar et al., combined with wireless pressure sensors, provides a new solution for blood pressure monitoring and thrombosis treatment [72].

(3) Signal accuracy and energy support optimization

By combining organic and inorganic materials, the Hittini et al. successfully realized a sensing system with high sensitivity, achieving a 100 ppb detection threshold and a 16.37 ± 1.42 s response time at 40 °C, significantly improving the perception accuracy and stability of e-skin [73]. Wang et al. further improved the measurement accuracy of static and dynamic forces by integrating piezoelectric components into the field effect transistor (FET) and promoted the application of electronic skin in mechanical sensing [74]. Nguyen et al. successfully distinguished the temperature and pressure signals in the FET sensor by using the bias method, achieving temperature sensitivity of 2.9 mV/°C and pressure sensitivity of 1.5 μA/kPa, improving sensing accuracy and reducing signal interference [75]. By integrating machine-learning and edge-computing technologies, MIT has developed intelligent algorithms that significantly improved real-time data analysis efficiency by 30% and sensor feedback accuracy by 25%, enabling complex decisions and real-time responses [76]. Research teams at the National University of Singapore and other universities are committed to developing and optimizing wireless communication protocols, thereby improving the efficiency and reliability of data transmission in sensor networks, ensuring data privacy and security, and preventing data leakage and abuse [77,78,79,80,81]. In terms of energy harvesting and sustainable solutions, the bio-fuel-powered soft e-skin developed by Mohammadifar et al. enabled multiple wireless sensing and provides sustainable energy support for the human–machine interface [82]. Tian et al.’s team explored sustainable power sources driven by both steam and heat, achieving energy outputs of 2.5 W/cm^2^ and 3.1 W/cm^2^, respectively, for portable electronic devices [83]. The asymmetric hygroscopic structure designed by Zhang et al. can be used for power generation and storage driven by moisture, providing a new idea for the development of environmental protection sensors [84]. Liu ’s team studied the application of microbial biofilm in electricity generation from water evaporation and the energy supply of wearable devices, thus promoting the recycling and environmental friendliness of electronic skin sensors [85].

These studies not only gradually break through the technical bottleneck of electronic skin but also lay the foundation for interaction between robots and humans. However, the current research on e-skin is less oriented towards environmental psychology and emotional interaction. From the perspective of human–computer interaction, electronic skin can be used as a new type of human–computer interaction interface, through the acquisition and decoding of electromyographic signals, to display dynamic patterns and text in specific scenes, provide visual feedback, realize interpersonal interaction and human–computer interaction, and bring new application prospects for wearable devices, health monitoring, human–computer interaction, and intelligent robots.

### 1.3. Electronic Interactive Tattoo

We propose that the skin, as the largest organ of the human body, has the potential to assume more complex functions and textures. By developing skin-like devices, we aim to facilitate more efficient communication between individuals. These devices could provide a novel means of communication and emotional expression for individuals with autism, and aphasia, and those in special scenarios where verbal communication is not possible. Furthermore, such innovations could pave the way for the development of interactive electronic tattoos that meet the demands of contemporary society.

Different countries and different times have different attitudes towards tattoos, and the historical culture and emotional spirit carried by tattoos have also changed. Additionally, people’s desires and fears about tattoos are constantly changing. Reducing the negative emotional color of tattoos not only depends on education but also on designers giving tattoos new characteristics, changing people’s cognition of tattoos and then accepting and developing them.

For deaf–mute, aphasia, autistic patients, affective communication disorder and other groups, due to congenital or acquired factors, have different communication disorders in verbal or non-verbal communication. Autistic patients, and affective communication disorder and other patients, may also have emotional and behavioral abnormalities [86]. These have greatly affected their social interactions [87]. People are social animals, and if they lose effective ways of socializing, they will feel lonely and socially isolated. Long-term loneliness and social isolation may lead to mental health problems, which may eventually lead to mental health problems such as depression and anxiety [88]. Secondly, social interaction can stimulate the brain and help maintain a person’s cognitive function [89]. A lack of social interaction can lead to cognitive decline. Compared with people with healthy social connections, people without social connections have a higher risk of cognitive decline. The 3-year ratio was 2.24 (95% CI, 1.40 to 3.58; *p* < 0.001), the 6-year ratio was 1.91 (CI, 1.14 to 3.18; *p* = 0.01), and the 12-year ratio was 2.37 (CI, 1.07 to 4.88; *p* = 0.03) [90]. At the same time, the flow of information, innovation, and cultural expression is limited by the lack of effective ways to socialize [91].

The rapid development of modern technologies, such as speech recognition and synthesis, and AI-assisted communication technologies can provide these groups with more communication options, help them better communicate with the world, and improve their independence and quality of life. It even provides a new means of expression for the psychological relief groups who need to be alone during the depression period so as to help them better adapt and recover. For people with autism, behavioral interventions such as applied Behavioral Analysis Therapy (ABA) and the Early Intervention Denver Model (ESDM) have been shown to improve communication skills and socialization [92,93].

Driven by the recognition of communication barriers faced by individuals with speech disabilities or in situations where verbal interaction is unfeasible or undesirable, we highlight the necessity for more inclusive and efficient communication methods. To address this need, we propose the Wearable Arduino-Based Electronic Interactive Tattoo (WABEIT). By leveraging the natural emotional fluctuations and subtle movements of the human body, WABEIT integrates sensors capable of detecting physiological responses, such as changes in muscle tone, which may correlate with an individual’s mood or intentions. Through pattern-switching mechanisms, WABEIT can intuitively convey the wearer’s real-time emotions and needs, thereby facilitating effective communication with the external environment.

## 2. System Requirements and Design

As an open platform that is simple and easy to learn, Arduino includes open-source hardware and software IDE 2.3.4 (Interaction Design Institute Ivrea), providing developers with unlimited possibilities. Arduino is relatively simple and easy to learn [94]. It is developer-friendly and suitable for designers and other non-professional programmers and embedded system developers. Therefore, the research team selected this platform to design and develop WABEIT, making the skin have the functions of color and luminescence, and connecting it with a single-chip microcomputer and sensors to respond in real time to multi-modal sensors, thereby achieving the emotional perception and expression of the skin.

### 2.1. Basis of the System Design

The microcontroller used by WABEIT is Arduino Nano R3. Arduino Nano is a development board similar to Arduino UNO R3, a kind of printed circuit board (PCB) with AVR microcontroller as the core controller. The DC power interface and voltage regulator circuit of Arduino Nano are removed. A mini-B standard USB socket is used [95]. Nano is based on the ATmega328P development board, which can be directly plugged into the breadboard for use (Table 1). Different sensors are enabled by powering the digital and analog pins of the device through programming languages derived from C and C++.

Vision accounts for 70% of the external information obtained by the human body [97]. Therefore, we have chosen to transform auditory elements into visual cues to assist individuals suffering from aphasia, thereby facilitating more effective communication and interaction. WABEIT designed in this research is a wearable display device with HCI function, which can interactively express the corresponding elements by collecting the user’s movements.

**Figure 2:**
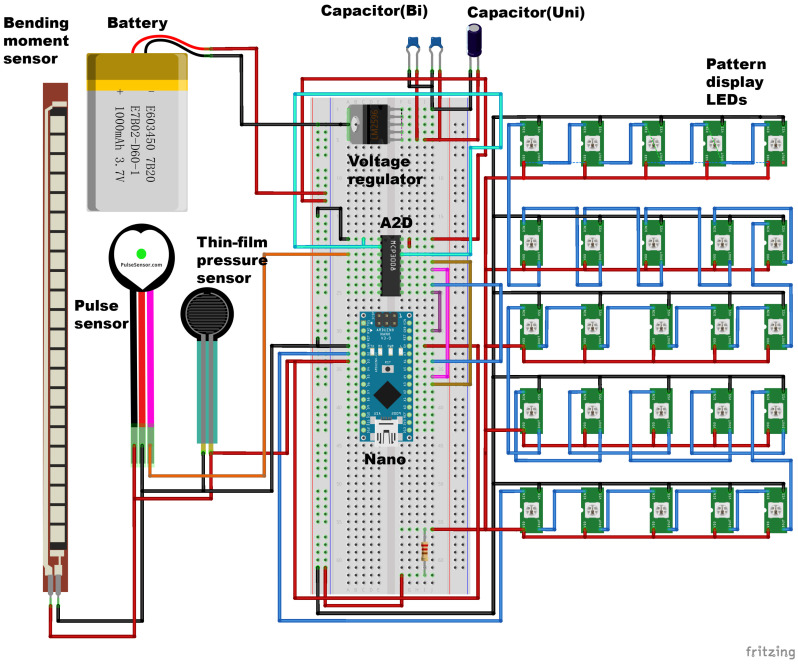
Electronic circuit of the WABEIT. WABEIT has the LEDs, pulse sensor, pressure sensor, and bending-moment sensor connected to the PWM ports. The availability of each component is confirmed in the overall function realization. Additionally, the voltage regulator is added to regulate the voltage of the power supply, and the A2D is used to realize the analog-to-digital conversion.

The inputs signal equipped with a heart rate sensor, a bending-moment sensor, and a film pressure sensor. The output of the system is an SMD LED matrix developed by the team, with polydimethylsiloxane (PDMS) serving as the base carrier. The LEDs are welded and packaged according to a specific circuit design and pattern. Users can apply the electronic tattoo by adhering it to the skin (Figure 2).

The Nano board has sufficient pins to do the job. It has 22 digital input/output pins, of which 6 can be used as PWM outputs, eight analog inputs, and one universal asynchronous receiver/transmitter (UART) hardware serial port [98]. WABEIT utilized SMD 5050 LED units, film pressure sensors, and bending-moment sensors, all of which are based on the Transistor–Transistor Logic (TTL) protocol and will connect to the digital pins. In the later stage, other universal asynchronous receiver/transmitter (UART)-protocol-based signal input sensors are also considered. We have determined the response time of the sensor and the SMDs (Table 2). Most importantly, the Nano’s size and weight are highly suitable for wearable applications. Weighing only 7 g, it can be comfortably and conveniently placed on the body without compromising comfort or causing the inconvenience associated with larger devices. The human–computer interaction (HCI) device based on the Nano facilitates low-cost, seamless interaction. Human interaction typically operates on a second-level scale. The response time precision of the Nano is at the millisecond level, which can be effectively combined with well-designed motion capture systems to achieve seamless HCI.

### 2.2. Choice of Hardware

WABEIT’s manufacturing budget is broken down into different components (Table 3). The total budget for the obligatory component of the base version of the project is USD 69.47. The cost of adding additional extensions will increase with the price of the required components, but will not exceed USD 150.

### 2.3. Open-Source Programming

**Figure 3:**
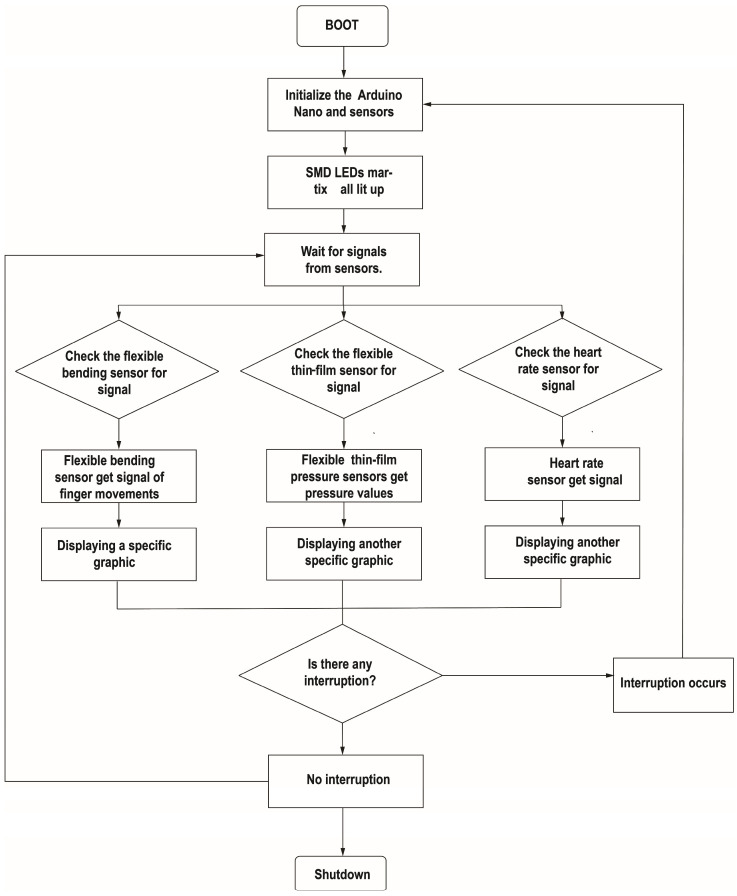
Flow chart of WABEIT.

The hardware programming of this device utilizes an integrated development environment (IDE), which is compatible with various platforms (Linux, Windows, and Mac) and can be freely used under the GNU General Public License. The programming language used by the device is derived from C and C++, and the flowchart of the program is as follows (Figure 3).

## 3. Functional Validation

In this study, the WABEIT was designed to provide novel interaction and communication solutions for individuals in specialized scenarios. Based on ergonomic standard data, we systematically evaluated the materials, structure, and functionality of the WABEIT. Through rigorous experiments and analyses, we determined the accuracy and error characteristics of the materials and sensors used in WABEIT. Additionally, we developed calibration and correction schemes to optimize the device’s performance across different environmental conditions.

### 3.1. Substrate Material

**Figure 4:**
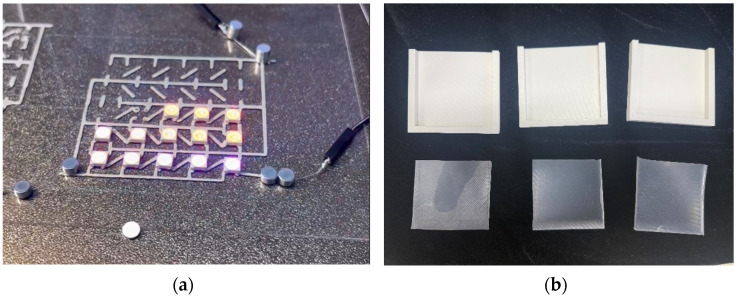
WABEIT with different substrate materials: (**a**) WABEIT circuit printed on PDMS; (**b**) WABEIT with silicone substrate material with thickness 1 mm, 1.5 mm, and 2 mm using 3D-printed molds.

The WABEIT developed in this study is used for visual communication between people; therefore, the size should not be too small. Then, an appropriate substrate material should be selected to carry and connect the electronic equipment. In the process of substrate material selection, we verified the performances of the PDMS material and silica gel in different proportions and thicknesses. Additionally, we utilized 3D printing technology to fabricate PDMS and silicone rubber molds, precisely controlling their size and thickness (Figure 4).

Polydimethylsiloxane (PDMS) is a permeable material that belongs to the category of polymer membranes. PDMS is non-porous and features excellent transparency, biocompatibility, elasticity, stretchability, temperature resistance, aging resistance, and compatibility with active materials [98]. PDMS has excellent chemical stability, which can maintain stable performance in the range of −60 °C to 200 °C. Additionally, it is resistant to chemical corrosion and can withstand the erosion of various solvents such as acid and alkali. It exhibits excellent chemical stability, maintaining stable performance over a temperature range of −60 °C to 200 °C. It is highly resistant to chemical corrosion and can withstand the erosion of various solvents, including acids and bases. The micro-structured PDMS film enhances the elastic deformation performance of the sensor while also providing higher sensitivity and faster response times compared to the unstructured PDMS film [99]. PDMS has a diffusion coefficient of 2000–4000 µm^2^/s for oxygen and 1000 µm^2^/s for CO₂. Based on these properties, PDMS-based electronic skins can be fabricated into porous structures with excellent air permeability, enabling the passage of air and water vapor and thereby enhancing wearability comfort [100].

Silicone is a highly active, semi-inorganic polymer composed of silicon dioxide, polysiloxane, silicone oil, coupling agents, fillers, and other inert compounds. It is insoluble in water and most solvents, odorless, tasteless, and chemically stable, reacting only with strong alkalis and hydrofluoric acid. With specific modifications, silicone can exhibit fire-retardant and anti-aging properties [101]. The unique combination of inorganic and organic structures within silicone molecules results in relatively longer atomic distances, providing high molecular flexibility. This characteristic enhances its gas permeability, allowing an easy passage of gas molecules [102]. As an adsorbent material, different manufacturing processes yield distinct micro-pore structures in various types of silicone. Its exceptional molecular structure grants it superior thermal stability, enabling it to maintain performance at temperatures as high as 120 °C for up to 20 years [103].

Both of the above two materials are suitable for use as the substrate material of electronic skin. Therefore, we will further conduct experimental verification on their safety, stretchability, and wearability. To evaluate the performance of the two materials at different hardness levels and thicknesses, we prepared 3D-printed molds with the same surface area but varying depths. For PDMS, we used a graduated cylinder to mix the PDMS base liquid with the curing agent at the required ratio for the experiment. The mixture was then degassed, poured into the mold, and left to cure naturally for 20 h before use (Figure 4b). For the silicone material, we purchased silicone with different hardness levels. The silicone A component is the base material, while the B component is the curing agent and catalyst. These were mixed at a volume ratio of 1:1, thoroughly stirred, degassed, poured into the mold, and demolded after 4 h for use. The dimensions of the PDMS and silicone test objects for WABEIT were set to approximately 60 mm×60 mm. Thickness is one of the conditions that need to be verified. Therefore, we selected PDMS (1:10) and silicone with a Shore hardness of 5° as the substrate materials for tensile strength verification. The experiment tested three material thicknesses: 1 mm, 1.5 mm, and 2 mm (Table 4).

The elongation after fracture δ was measured for the experimental samples’ stretchability. Before the tensile test, the initial gauge length $L_{0}$ of the specimens was recorded. After fracture, the final gauge length $L_{k}$ was measured. The elongation after fracture was then calculated using the equation:

(1) $$\delta =\frac{L_{k}-L_{0}}{L_{0}}\times 100\%$$

Under tensile testing, the PDMS with the 1 mm thickness sample was stretched from an initial length of 60 mm to 82.49 mm, δ≈37.48%; the 1.5 mm thickness sample was stretched from an initial length of 60 mm to 87.98 mm, δ≈46.63%; the 2 mm thickness sample was stretched from an initial length of 60 mm to 86.35 mm, δ≈43.92%. Within the control group, the silicone with the 1 mm thickness sample was stretched from an initial length of 60 mm to 114.79 mm, δ≈91.32%; the 1.5 mm thickness sample was stretched from an initial length of 60 mm to 104.27 mm, δ=73.78%; the 2 mm thickness sample was stretched from an initial length of 60 mm to 107.69 mm, δ=79.48% (Table 5). Though the silicone may excel in terms of stretchability, PDMS offers superior stability and insulation properties. The results demonstrated that the tensile strength was optimal at a thickness of 1.5 mm, indicating that increased thickness does not necessarily enhance tensile strength.

Subsequently, at a thickness of 1.5 mm, we compared the rigidity of PDMS samples prepared with different curing agent-to-base adhesive ratios, specifically 1.5:10, 1:10, and 1:20. The base adhesive and curing agent were mixed in the prescribed ratio, degassed to remove bubbles, and then poured into a 3D-printed mold. The mixture was subsequently cured on a heating platform at 60 °C for 10 h. After curing, the PDMS samples were demolded and subjected to tensile testing.

The results revealed distinct differences in material rigidity under these varying hardness ratios (Table 6). The PDMS with the 1.5:10 ratio sample was stretched from an initial length of 60 mm to 87.97 mm, δ≈46.62%; with the 1:10 ratio sample was stretched from an initial length of 60 mm to 74.92 mm, δ≈24.87%; with the 1:20 ratio sample was stretched from an initial length of 60 mm to 77.92 mm, δ≈29.87% (Table 7). In tensile tests conducted across three different curing agent-to-base adhesive ratios, the 1.5:10 ratio exhibited the highest mechanical stability.

Based on these results, we selected PDMS with a thickness of 1.5 mm and a curing agent-to-base adhesive ratio of 1.5:10 as the substrate material for WABEIT.

### 3.2. Comparison of Conductive Materials

The resistivity, purity, and mechanical and thermal stability of conductive materials, among other factors, collectively determine the transmission efficiency of circuits. Selecting appropriate conductive materials for fabricating WABEIT is crucial for the success of the device. Three types of conductive materials were selected for fabricating WABEIT in this study: silver ink, electrically conductive ink, and silver wires (Table 8).

Silver ink has demonstrated excellent electrical conductivity, strain sensing ability, electric heating capability, low driving voltage, rapid response, and high flexibility, making it a promising candidate for wearable electronic devices [104]. In this study, the SP-7011 model silver ink was selected. This stretchable conductive silver ink is suitable for flexible circuit printing on elastic films and textile fabrics. After complete curing at an appropriate temperature, it exhibits superior electrical conductivity, low resistance, and good flexibility, hardness, and oxidation resistance. However, silver particles in the paste may migrate under certain conditions, such as high humidity, leading to circuit short-circuiting or performance degradation. The preparation of conductive silver paste requires strict control of component ratios, stirring time, and temperature to ensure optimal performance.

Electrically conductive inks are paste-like materials composed of conductive substances (such as gold, silver, copper, and carbon) dispersed in a binder and are commonly used for printed circuits, electrodes, electroplating substrates, keyboard contacts, and printed resistors [105]. In this study, Elasink 990M, a conductive material primarily composed of a gallium–indium alloy, was selected for its stretchable properties and suitability for flexible circuit printing on elastic films and textile fabrics. After drawing the circuit, the material was cured at 80 °C for 10 min to achieve optimal performance.

Silver wires exhibit high electrical conductivity, stretchability, and strong adhesion, making them suitable for applications in wearable electronics. Compared to other materials, silver wires possess low electrical resistivity, which enhances signal fidelity. In wearable devices, such as those incorporating multiple material interfaces, silver wires ensure stable electrical connections and reduce signal interruptions or transmission instability caused by poor contact. The elongation at the break of silver wires can reach 20–30% [106], allowing them to bend and stretch without breaking easily. This property enables silver wires to adapt to human body movements, making them ideal for flexible wearable devices in various scenarios.

**Figure 5:**
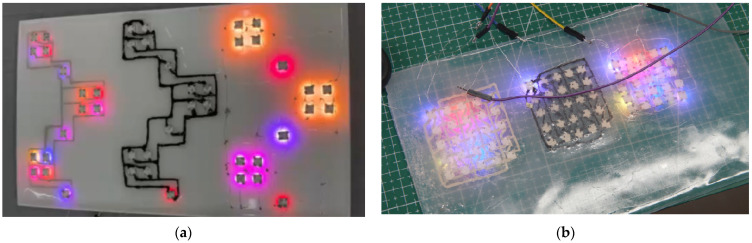
WABEIT with different circuits on PDMS substrates: (**a**) In each circuit, 15 SMDs were connected in series. The conductive materials used were silver ink, electrically conductive ink, and silver wire, arranged from left to right; (**b**) 25 SMDs were connected in series in each circuit. The conductive materials used were silver ink, electrically conductive ink, and silver wire, arranged from left to right.

In the experiment, silver ink and electrically conductive ink were used as printing materials with a 2D plotter to fabricate electronic tattoo pattern circuit on PDMS substrates. The final patterns exhibited slight differences due to the varying adhesion properties of the materials. We fabricated the same electronic tattoo circuit on PDMS with 0.3 mm diameter silver wire as the circuit material. After completing the circuit, 15 SMD LEDs were connected inside each circuit. These circuits were powered by an Arduino Nano microcontroller, which also provided the input signal. The final display is shown in Figure 5.

Experimental results indicated that the conductive ink exhibited poor performance, failing to support the simultaneous illumination of multiple LED beads. In contrast, both silver paste and silver wire circuits were capable of lighting all 15 LED beads. However, the brightness of the silver paste circuit was significantly lower than that of the silver wire circuit.

To further investigate the suitability of the three materials for accurately transmitting electrical currents and signals during human body movements, we conducted tensile conductivity experiments. We applied conductive materials onto the 1.5 mm thick PDMS substrate to create conductive layers and established conductive circuits on the PDMS samples. SMD5050 LEDs were positioned centrally and connected to an Arduino UNO board via fixtures or probes to measure during the stretching process. The samples were securely mounted in the fixture of a digital force gauge to ensure they remained stable throughout the stretch. The gauge was then used to incrementally apply tensile force, while we monitored the deformation of the conductive material.

**Figure 6:**
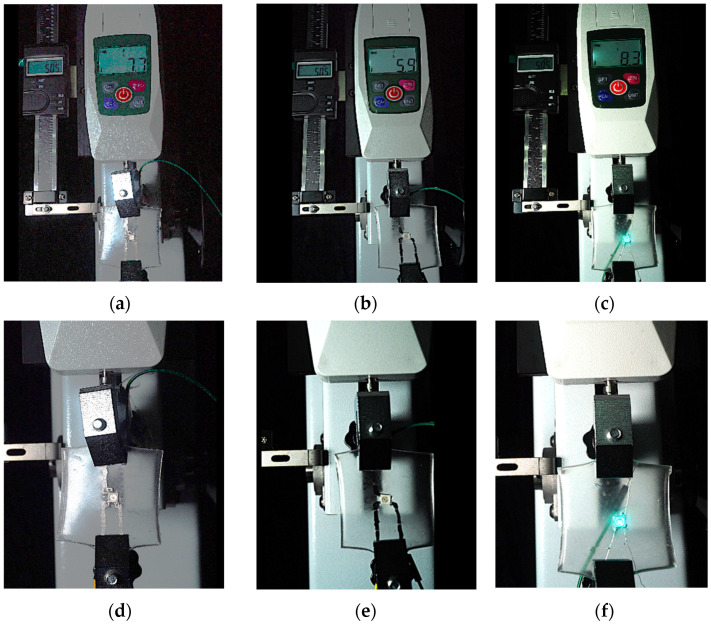
Electronic tattoo pattern circuit on PDMS substrates with different materials: (**a**) silver ink circuit ruptured at a tensile extension of 5.05 mm; (**b**) conductive ink ruptured at a tensile extension of 5.05 mm; (**c**) silver wire circuit maintained its conductivity at a tensile extension of 5.05 mm; (**d**) silver ink ruptured details; (**e**) conductive ink ruptured details; (**f**) silver wire details.

In the experimental investigation, we observed the electrical conductivity of conductive materials on a 50 mm PDMS substrate under tensile stress using a digital push–pull force gauge. The silver paste circuit was subjected to a tensile force of 7.7 Newtons, and conductivity was lost when the tensile strain reached 55.05 mm, accompanied by the formation of minor transverse cracks. In the case of the conductive ink circuit, a 5.9 Newton tensile force was applied, and conductivity ceased at the same strain level of 55.05 mm, with the circuit exhibiting significant multiple cracks. Notably, the silver wire circuit maintained its conductivity when pulled to 55.05 mm under an 8.3 Newton tensile force (Figure 6).

Therefore, this project opted to use 0.3 mm silver wire as the material for the electronic tattoo circuit.

### 3.3. Pattern Display Material

In this study, we selected the SMD 5050 LED lamp beads for its programmable RGB display capabilities. This LED features a compact size of 5.0 mm×5.0 mm×1.6 mm and can display up to $2^{24}$ colors with a data transmission speed of 800 kbps [107]. It operates at a low power consumption of 0.18 W and has a wide operating temperature range of −35 °C to +60 °C. The LED is composed of non-toxic materials, unlike mercury-containing fluorescent lamps, and exhibits high brightness with minimal thermal radiation compared to HID or incandescent lamps. Under optimal current and voltage conditions, its service life can reach up to 100,000 h. Encapsulated in epoxy resin, the LED is more durable than traditional light bulbs or fluorescent tubes.

The forward voltage of the 5050 LED typically ranges from 2.8 V to 3.3 V, with a maximum current of 20 mA and a maximum brightness of 30 lm. However, this maximum brightness can cause visual discomfort or even damage to the human eye. For optimal visual comfort under normal reading conditions, the ambient light brightness should be above 300 lx. Based on relative brightness estimates, the suitable brightness range for the 5050 LED when used in close proximity to the human eye is approximately 10% to 30% of its maximum brightness [108]. Therefore, in this experiment, the brightness of the wearable electronic device was controlled within the 10–30% range to ensure eye safety, corresponding to a current of 2–6 mA.

**Figure 7:**
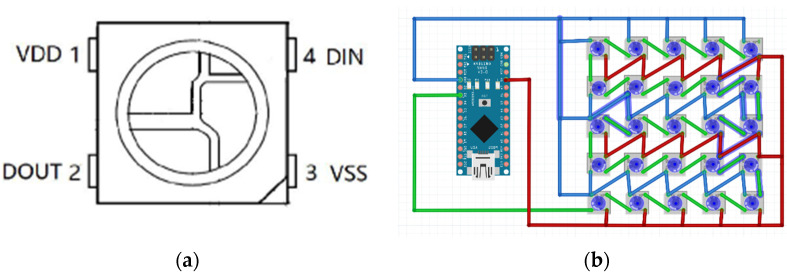
(**a**) Schematic diagram of the SMD 5050; (**b**) circuit layout design scheme.

To ensure the voltage and current stability of this component, we calculate whether WABEIT needs external capacitors and resistors (Figure 7). The formula is as follows:

(2) $$R=\frac{V_{cc}-V_{F}}{I_{F}}$$

$V_{cc}$ is the supply voltage and the output voltage of Arduino Nano is 5 V.

$V_{F}$ is the forward voltage of the LED, and the forward voltage of the common positive RGB lamp beads selected by WABEIT is 3 V.

$I_{F}$ is the rated current of the LED, assuming that the current of a single 5050 lamp bead is 4 mA, and 25 beads are 100 mA.

So, R=20 Ω, which can be achieved in this experiment by using the Nano microcontroller with its own pull-up resistor.

The choice of capacitance depends on the following calculation formula:

(3) $$C=\frac{I_{F}}{∆V\times f}$$

- $I_{F}$ is the load current and is calculated with a maximum current of 20 mA × 25 lamp beads.
- ∆V is the ripple voltage, and WABEIT requires the current to be controlled at 2–6 mA; therefore, the ripple voltage is set to 0.1 V.
- f is the power supply frequency, which is assumed to be 50 Hz.

So, C = 1000 μF. As determined by calculation, an electrolytic capacitor of 1000 μF is connected in parallel at the WABEIT input to reduce power ripple and voltage fluctuation.

### 3.4. Verification of Thin-Film Pressure Sensor

WABEIT is designed to enable wearers to express emotions in specific situations. Many individuals exhibit *tension-related gestures*, such as fist clenching and finger picking, when experiencing significant psychological stress. To address this, we integrated a pressure sensor into the device, allowing wearers to convey their emotional states to the outside world through subtle and involuntary pressure-relief actions. This design aims to enhance understanding and promote stress relief. The calibration of the thin-film pressure sensor was conducted in real-world office and living environments. In this study, we compared three types of thin-film pressure sensors: RP-C7.6-ST, RP-C7-LT, and FSR400 (Table 9).

**Figure 8:**
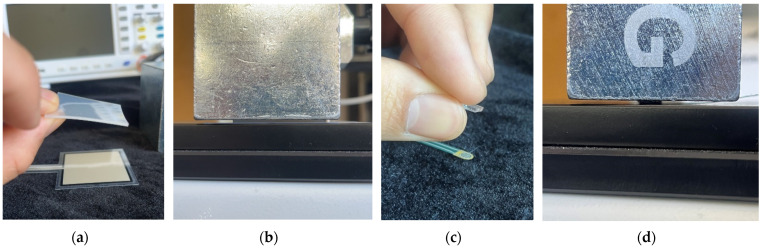
The PI module assists the pressure sensor in receiving pressure from all the lead blocks. (**a**) PI module placed on FSR400; (**b**) waveform experiment when the lead blocks transmit gravitational force to the FSR400 via the PI module; (**c**) PI module placed on RP-C7-LT; (**d**) waveform experiment when the lead blocks transmit gravitational force to the RP-C7-LT via the PI module.

To simulate the pressing actions of human hands on the thin-film pressure sensor with varying strength levels, three 1 kg lead blocks were used to gradually apply and release pressure. The resulting waveform was captured using a FNIRSI-1014D oscilloscope (Table 10). We placed a 2 mm thick polyimide module (PI) on each piece of the thin-film pressure sensor to ensure that all the lead block pressures were transmitted to the sensor (Figure 8).

The readings of voltage division ($V_{div}$), time division ($T_{div}$), peak-to-peak voltage ($V_{pp}$), average value ($V_{avg}$), and frequency (f) on the oscilloscope are listed below (Table 11).

The oscilloscope enables a clear comparison of the response sensitivity and output amplitude of the two sensors under identical pressure conditions. Sensors with higher sensitivity, characterized by larger output signal changes under the same pressure, are more suitable for detecting subtle human movements. Additionally, the consistency and stability of the sensor output were evaluated by repeatedly applying and releasing the same pressure. Sensors exhibiting good repeatability and stable signals are more reliable for long-term applications. Furthermore, the response time, defined as the interval from pressure application to output signal stabilization, and the recovery time, from pressure release to signal recovery, were measured using rapid application and removal of a lead block. Shorter response and recovery times indicate superior dynamic performance, enabling the sensor to more effectively capture transient small motions.

To systematically compare the response times of the three sensors, pairwise comparisons were conducted. The outputs of two sensors were connected to the two channels of an oscilloscope, and rapid applications and releases of lead blocks were employed to simulate step pressure changes. The signal changes on the oscilloscope channels were observed to determine the response and recovery times of each sensor pair. Through this comparative analysis, it was found that the RP-C7-LT sensor exhibited the shortest response and recovery times. Consequently, the RP-C7-LT was selected as the signal-generating sensor for detecting tension-related gestures on the wearer’s hand.

### 3.5. Verification of Bending-Moment Sensor

Finger movements are an integral part of non-verbal behavior and can serve as a reliable indicator of an individual’s emotional state. In the context of counseling and clinical psychology, the observation of finger movements provides professionals with valuable insights into a client’s emotional status. When individuals experience stress or frustration, their emotional states are often manifested through non-verbal cues, including specific finger movements. For instance, under conditions of nervousness, fingers may exhibit trembling, tapping, or clenching. Conversely, when individuals are frustrated, their fingers may appear weak and sluggish. These subtle yet significant gestures can reflect underlying psychological states and are crucial for understanding the complex interplay between emotions and non-verbal expression [109].

Therefore, we chose bending-moment sensors, Flex4.5, to fit on the wearer’s finger to monitor the wearer’s finger movements (Table 12). To evaluate the sensitivity of the Flex4.5 sensor for monitoring finger movements, we conducted a series of experiments. The sensor was attached to the right index finger of a subject, and various degrees of finger bending were simulated, including slight bending (approximately 30°), mild bending (approximately 45°), moderate bending (approximately 80°), and severe bending (>90°). These movements were designed to mimic the dynamic changes in finger movements during daily activities.

The signal change curves of the Flex4.5 sensor were recorded during these bending actions (Table 13). The results indicated that the Flex4.5 sensor exhibited short response and recovery times, demonstrating its ability to rapidly capture the dynamic changes in finger movements. This high sensitivity makes the Flex4.5 sensor particularly suitable for monitoring subtle finger movements, which are often associated with fine motor tasks and emotional states.

### 3.6. Heart-Rate Pulse Sensor

When experiencing emotional fluctuations such as tension or anger, the human body initiates a stress response, which typically results in a significant increase in heart rate. This physiological change is a natural response to stress, aimed at providing the body with more oxygen and energy to cope with potential dangers or challenges. The resting heart rate of adults’ ranges from 60 to 100 beats per minute, while under stress, it may increase to 120 beats per minute or higher [110]. Based on these considerations, we developed a device to provide an alternative emotional expression solution for individuals with aphasia. The device monitors the user’s real-time heart rate and provides feedback through an electronic tattoo pattern.

In this study, a pulse sensor was selected as the signal acquisition device for heart rate detection, and a wearable smartwatch with heart rate monitoring capabilities was used as a comparison (Table 14). The Apple Watch’s heart rate monitoring feature, including its atrial fibrillation (AFib) detection, has been approved by the US Food and Drug Administration (FDA) as part of the Medical Device Development Tool (MDDT) project. Several studies have demonstrated the high accuracy of the Apple Watch’s heart rate monitoring, with an accuracy rate of up to 92% in detecting AFib [111].

**Figure 9:**
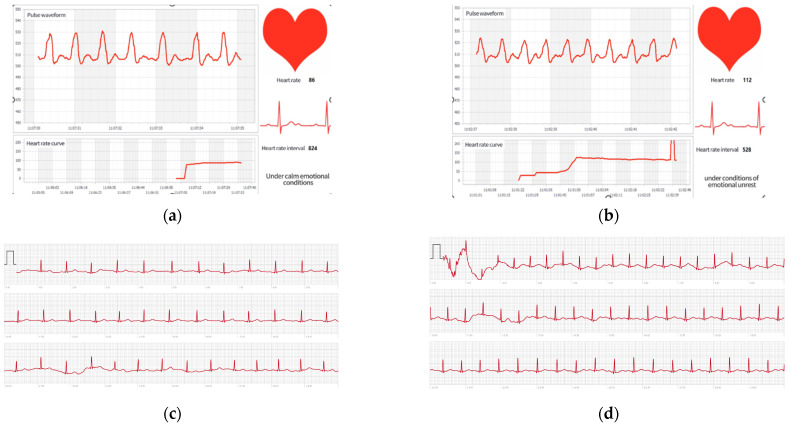
Heart rate data measurement in resting and active states: (**a**) heart rate data in resting states using pulse sensor; (**b**) heart rate data in active states using pulse sensor; (**c**) heart rate data in resting states using Apple Watch (The grey font in the figure is the timing unit second); (**d**) heart rate data in active states using Apple Watch (The grey font in the figure is the timing unit second).

In this experiment, we compared the heart rate data obtained from a pulse sensor and an Apple Watch. Specifically, the pulse sensor data were collected using Arduino software and a Nano board. The collected data were then exported to a host computer for further analysis. Simultaneously, the Apple Watch was employed to collect heart rate data from the human subject, and the corresponding data were exported for comparative analysis. This dual-method approach allowed us to evaluate the consistency and accuracy of heart rate measurements between the two devices under identical conditions (Figure 9).

**Figure 10:**
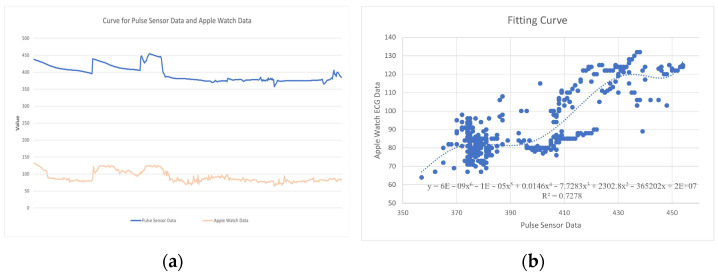
Correlation analysis on 405 sets of data: (**a**) pulse sensor data and Apple Watch data; (**b**) linear regression method to fit and generated a fitting curve.

We wear the pulse sensor and Apple Watch simultaneously on the left middle finger and left wrist of the experimenter, perform different body movements to change the heart rate, and conduct correlation analysis on 405 sets of data (Figure 10).

It was found that the data collected by the pulse sensor and Apple Watch data were not completely linearly correlated so we used the *linear regression* method to fit the two sets of data, generated a fitting curve, and generated a sixth-degree polynomial fitting formula as follows:

(4) $$y=6E-{09X}^{6}-1E-{05X}^{5}+{0.0146X}^{4}-{7.7283X}^{3}+{2302.8X}^{2}\;-365202X+2E+07$$

We will use this formula to correct the data acquired by the pulse sensor and use this data as the input signal to let WABEIT generate interactive feedback.

## 4. Discussion

To evaluate the practicality of WABEIT in emotional expression and communication for individuals with aphasia, we conducted a series of experiments by wearing the device on a human model across various scenarios. The results demonstrated efficient and accurate data collection, with the tattoo pattern’s design, color, and brightness exhibiting high reliability. This study highlights the potential of electronic skin to integrate sensors that detect physiological responses and emotional states, thereby facilitating diverse and multi-scenario communication for the aphasia community. Our findings provide a foundation for further exploration of specialized sensors and the development of new applications in this domain.

### 4.1. Input and Output Settings

Based on observations of group behavior, we designed communication scenarios for non-verbal emotional expression using electronic tattoos, as follows: (1) increasing pressure through hand pressing; (2) continuous finger bending to indicate emotional changes; and (3) significant changes in heart rate associated with emotional fluctuations.

**Figure 11:**
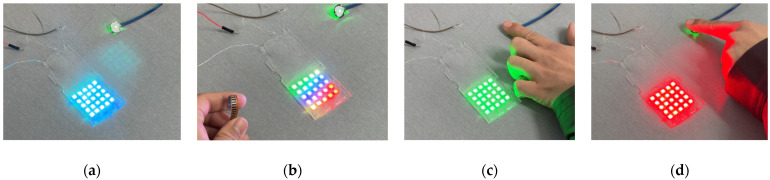
Four types of WABEIT displays: (**a**) no signal as user in calm status; (**b**) finger bending trigs color-running light pattern; (**c**) green light when user’s heart rate is low; (**d**) red light when user’s heart rate is high.

To meet user needs, the electronic tattoo patterns were categorized into four types: (1) calm: a blue breathing light continuously illuminates when the user is calm, with no pressing or finger-bending movements; (2) pressing: a rotating purple arrow indicates finger and palm pressing actions; (3) finger bending: a color-running light pattern represents bending movements, such as fist clenching, with the flashing frequency varying according to the pressure applied; (4) heart rate changes: all lights display green when the user’s heart rate is low. When the heart rate increases, all lights flash alternately in red and blue, with the frequency increasing in proportion to the heart rate (Figure 11).

These design strategies enable electronic tattoos to serve as an alternative communication tool for individuals with aphasia, facilitating diverse and multi-scenario interactions.

### 4.2. Results

**Figure 12:**
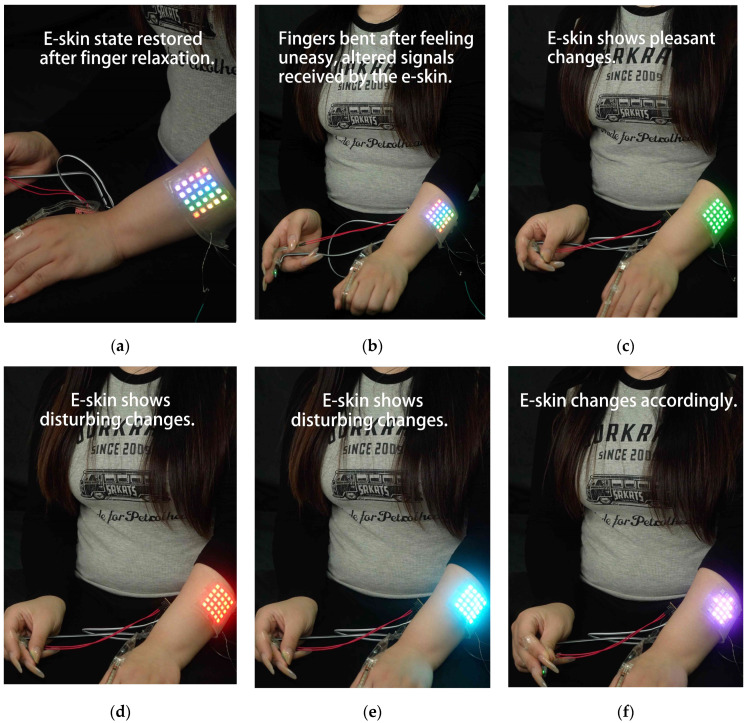
The participant was fitted with WABEIT devices for the experiment. (**a**) Rainbow light mode continues to light in normal state; (**b**) color-running light pattern when user bending finger; (**c**) green light when participant’s heart rate is low; (**d**) red and blue lights alternate when participant’s heart rate is high; (**e**) red and blue lights alternate when participant’s heart rate is high; (**f**) purple arrow rotates when participant presses the pressure sensor.

The sensitivity and responsiveness of the WABEIT were evaluated through experiments involving various stimuli from wearers. The thin-film pressure sensor integrated into the WABEIT demonstrated high sensitivity to mechanical pressure, with a rapid response time and recovery time that were imperceptible to the naked eye. Specifically, in practical wearing experiments, the system was programmed to wait 1 s after recognizing an action before providing feedback. This design ensured that the real-time monitoring capability for physical interactions was robust, highlighting the device’s ability to capture and respond to dynamic stimuli in real time (Figure 12).

To assess the portability and long-term usability of the WABEIT, the device was equipped with a mobile power supply and tested for continuous operation over a period of 2.5 h. During this test, the mobile power supply provided sufficient energy to support the device’s interactive functions, including sensor data processing and graphical display. The overall performance of the WABEIT remained stable throughout the test duration, with no significant power loss or performance degradation observed. This finding confirms that the WABEIT is well suited for wearable applications, ensuring both portability and long-term usability. The results suggest that the integration of a mobile power supply effectively addresses the challenges of power consumption and operational stability, thereby enhancing the practicality of the device for real-world applications.

Furthermore, the heart rate sensor embedded in the WABEIT exhibited high accuracy in detecting physiological signals. The mean absolute error of the heart rate measurements was less than 2 bpm when compared to a commercial heart rate monitor, indicating the device’s reliability in capturing physiological data. These results collectively highlight the WABEIT’s ability to effectively sense and respond to both environmental and physiological stimuli, thereby enhancing its potential for real time, interactive applications.

We further reviewed the design concept and implementation strategies of WABEIT with a view to achieve good application prospects on other wearable devices. WABEIT used a modular design that combines skin-friendly materials, multimodal sensing components, and embedded systems to successfully construct a wearable health monitor prototype. This prototype demonstrates similar sensitivity and interaction capabilities, verifying that WABEIT’s design approach can be generalized to other wearable applications.

In summary, the results of this study showed that WABEIT is highly sensitive, adaptable, portable, and socially acceptable. These findings highlight the potential of wearable interactive tattoos as a novel platform for human–computer interaction and personalized expression while also providing a basis for the development of other smart wearable devices.

## 5. Conclusions

To address the limitations of traditional electronic skin tattoos, such as inconvenient use, high time cost, negative body perception, and the lack of intelligent interaction, we designed and developed a novel wearable interactive tattoo on electronic skin (WABEIT). The WABEIT features a skin-friendly PDMS substrate integrated with multimodal sensing components, including a silver wire circuit, a programmable surface-mount device (SMD), a thin-film pressure sensor, and a heart rate sensor. Additionally, the device is equipped with an embedded Arduino Nano development board to enable intelligent interaction and is powered by a mobile power supply to ensure portability. Experimental results demonstrate that the WABEIT exhibits highly sensitive signal response, a wide range of pattern changes, and reliable interaction capabilities. Collectively, these features highlight its potential as a versatile and user-friendly wearable device, offering significant advantages over traditional electronic skin tattoos.

The WABEIT offers several significant advantages over traditional tattoos and other wearable devices. By utilizing a PDMS-based material, the WABEIT provides a non-invasive and skin-friendly alternative to traditional tattoos, thereby eliminating the pain, allergy risks, and lengthy production processes associated with conventional tattooing methods. The device is capable of dynamically displaying a wide range of colors and patterns in response to different needs and scenarios. This flexibility allows for effective expression of emotions and information. Additionally, the WABEIT features high brightness and relatively low power consumption, ensuring clear and bright visual effects while extending the device’s battery life. The WABEIT can dynamically adjust its display mode based on sensor inputs, providing users with intuitive visual feedback. This capability enables the device to adapt to various user needs, generate real-time feedback, and effectively express personal emotions, thereby enhancing user engagement and satisfaction. In the future, the e-skin could be integrated with other interaction modalities, such as touch and sound, to form a multimodal interaction system. This would provide richer information and facilitate more comprehensive user understanding and expression. Equipped with a mobile power supply, the WABEIT is ensured with continuous and stable operation over extended periods. This feature supports long-term wearability and maintains the device’s performance, making it highly suitable for practical applications in daily life.

Despite its promising results, the current study has several limitations that warrant further investigation. For example, the long-term durability and reliability of WABEIT under continuous use and exposure to environmental factors need to be evaluated through more extensive testing. When verifying the base material in this experiment, four-way stretching should be performed to simulate skin ductility. However, due to equipment limitations, only one-way stretching was conducted. Regarding the conductive materials, silver paste, graphite screen printing, and 3D printing were explored in this study, with silver wire ultimately being used for the final solution. Future production of WABEIT should consider more intelligent approaches, such as increasing the silver content in the silver paste or modifying the paste’s viscosity. Furthermore, the bending-moment sensor used in this study was limited to a single sensor worn on the index finger. However, human hand movements are diverse, with different fingers exhibiting varying sensitivity and ranges of motion. The combined movements between fingers are also worth further analysis as correlating these movements with human psychological states and emotions could activate more input signals and enhance the device’s functionality. Future work should focus on developing a multimodal interaction system that integrates touch, sound, and other modalities to provide richer information and improve user experience.

We envision that this device will significantly enhance users’ ability to communicate through diverse emotional expressions. Future work will focus on leveraging multimodal data from the device, such as monitoring body temperature, skin conductivity, and other physiological signals, to more accurately capture and differentiate wearers’ emotions, including happiness, sadness, and anger. By integrating these signals with dynamic changes in movement and heart rate, the device aims to provide more precise and context-aware emotional expressions. Additionally, we plan to expand the device’s visual capabilities by incorporating a wider range of colors and patterns to better convey complex emotions and enhance user engagement. To further improve the functionality and usability of the device, we aim to integrate wireless communication capabilities, enabling simple and intuitive group interactions through wireless network transmission. This feature will allow users to share emotional states and information seamlessly, fostering more effective communication and social interaction. In terms of energy sustainability, future development will explore the use of triboelectric and piezoelectric effects to convert mechanical energy into electrical energy, thereby achieving self-powered operation. This approach will eliminate the limitations of traditional rigid power sources, enhancing the device’s portability, flexibility, and long-term usability. Furthermore, we plan to increase the visible area of the electronic skin component to improve its visibility and effectiveness. The design will also accommodate personalized needs, allowing for non-matrix patterns that better align with wearers’ preferences. These advancements will not only enhance the device’s functionality but also provide a more natural and intuitive user experience, paving the way for the next generation of wearable interactive technologies.

In summary, WABEIT represents a significant advance in wearable technology, offering a unique combination of functionality, adaptability, and user-friendliness. Its potential for generalization to other wearable devices underscores its importance as a new platform for human–computer interaction and electronic applications. Future research will continue to explore and refine this technology, aiming to address existing limitations and unlock new possibilities for wearable electronics.

## Figures and Tables

**Table 1:** Technical parameters of Arduino Nano [96].

Nano Specification Parameter	
Operating voltage	5 V
Input voltage	7–12 V
Micro controller	ATmega328P
Input voltage (limit)	6–20 V
Digital I/O pins	16
Analog I/O pins	8
PWM pins	6
DC I/O pin	40 mA
DC for 3.3 V pin	50 mA
Flash memory	32 KB
SRAM	2 KB
EEPROM	1 KB
Clock frequency	16 MHz
Length	45 mm
Width	18 mm
Weight	7 g

**Table 2:** Response time of the sensor and the SMDs.

Unit	Type	Model	Response Time
PCB	Arduino	Nano	1–10 ms
LED	SMD	5050	1–5 ms
Input sensor	Pulse sensor	JXINW	50–150 ms
Input sensor	Film pressure sensor	Waaax	1–5 ms
Input sensor	Bending-moment sensor	Flex4.5	1–3 ms
Conductive materials	Silver wire	Φ 0.3 mm	<1 ms
Conductive materials	Silver ink	BroadCON-Ink 500	<1 ms
Conductive materials	Electrically conductive ink	Elasink 990 M	<1 ms

**Table 3:** Components of WABEIT.

Unit	Type	Model	Price
PCB	Arduino	Nano	USD 8.00
LED	SMD	5050	USD 0.12
Input sensor	Pulse sensor	JXINW	USD 15.00
Input sensor	Film pressure sensor	Waaax	USD 5.00
Input sensor	Bending-moment sensor	Flex4.5	USD 3.80
Conductive materials	Silver wire	0.3 mm	USD 3.08/m
Conductive materials	Silver ink	BroadCON-Ink 500	USD 2.00/m
Conductive materials	Electrically conductive ink	Elasink 990M	USD 50.00/kg
Substrate material	PDMS	Dow Corning	USD 35.00/kg
Substrate material	Silicon	Dow Corning	USD 30.00/kg

**Table 4:** The elongation comparison tests of PDMS sheets and silicone sheets.

	1 mm	1.5 mm	2 mm
PDMS	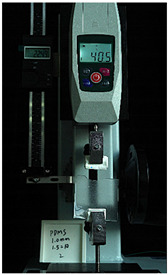	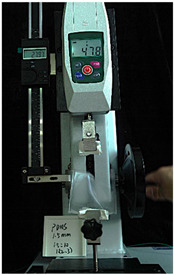	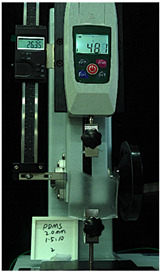
	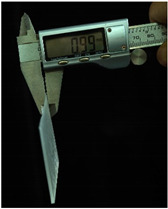	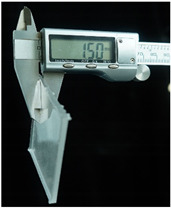	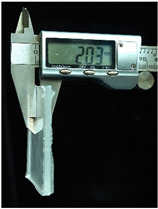
Silicone	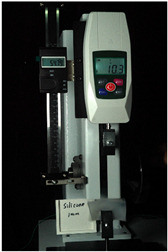	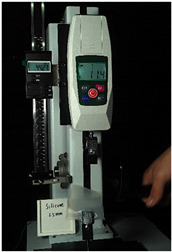	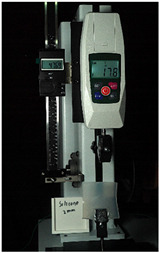
	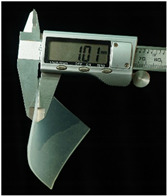	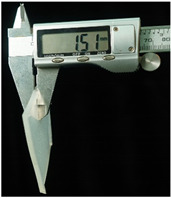	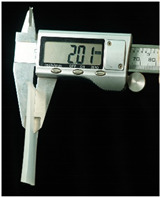

**Table 5:** Tensile test results.

	Thickness (mm)	Initial Length (mm)	Maximum Length (mm)	Elongation After Fracture δ (%)
PDMS	1	60	82.49	37.48
	1.5	60	87.98	46.63
	2	60	86.35	43.92
Silicone	1	60	114.79	91.32
	1.5	60	104.27	73.78
	2	60	107.69	79.48

**Table 6:** The elongation comparison tests of PDMS sheets in with different curing ratios.

1.5:10	1:10	1:20
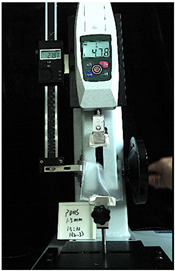	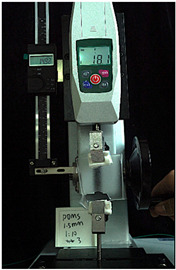	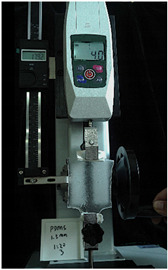

**Table 7:** Tensile test results with different adhesive ratio.

	Ratio	Initial Length (mm)	Maximum Length (mm)	Elongation After Fracture δ (%)
PDMS	1.5:10	60	87.97	46.62
	1:10	60	74.92	24.87
	1:20	60	77.92	29.87

**Table 8:** Comparison of conductive materials.

	Silver Ink	Electrically Conductive Ink	Silver Wires
Type	BroadCON-Ink550	Elasink 990M	--
Color	Silver Gray	Black Gray	Silver
Conductivity	$\sim 4\times 10^{7}\;S/m$	$\sim 0.8\times 10^{7}\;S/m$	$\sim 6.3\times 10^{7}\;S/m$
Viscosity	$\sim 3\times 10^{4}$ cps	$3\times 10^{4}$ cps	--
Fineness	≤20 μm/mil	≤60 μm	10.49 g/cm ^3^
Concentration	≥30 wt%	≥50 wt%	≥99.99%
Drying conditions	Oven 150 °C/30 min	Oven 150 °C/20 min	--

**Table 9:** Thin-film pressure sensors.

	RP-C7.6-ST	RP-C7-LT	FSR400
	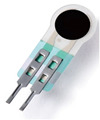	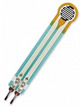	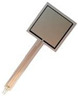
Range	30 g–1.5 kg	2 g–1.5 kg	50 g–2 kg
Operating temperature	−20–65 °C	−20–65 °C	−40 °C–85 °C
Recovery times	≤0.01 s	≤0.01 s	≤0.01 s
Response time	≤10 ms	≤3 ms	≤3 ms
Operating voltage	3.3–5 V DC	3.3–5 V DC	3.3–5 V DC
Resistance range	400 Ω–1 MΩ	100 Ω–10 MΩ	300 Ω–10 MΩ
Weight	1 g	1 g	1 g
Dimensions	14 mm × 32 mm × 1 mm	44 mm × 7 mm × 0.3 mm	40 mm × 40 mm × 0.46 mm
Durability	≥1 million cycles	≥1 million cycles	≥1 million cycles

**Table 10:** Waveform experiments for sensors underwent gravity-induced stress.

	1 kg	2 kg	3 kg
RP-C7.6-ST	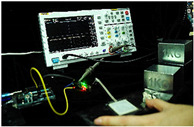	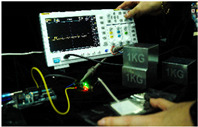	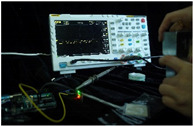
	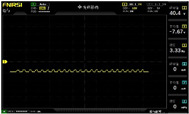	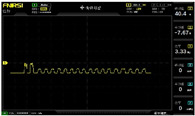	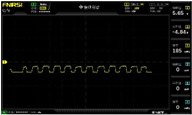
RP-C7-LT	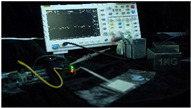	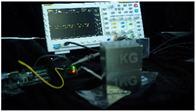	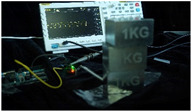
	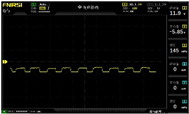	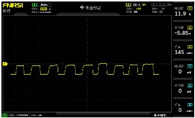	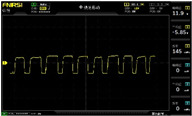
FSR400	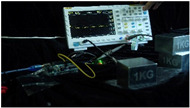	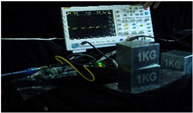	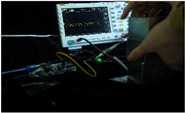
	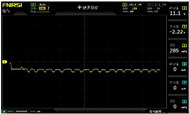	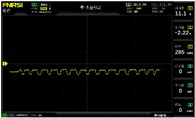	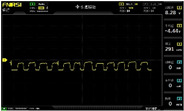

**Table 11:** Waveform results for sensors underwent gravity-induced stress.

	$V_{div}$	$T_{div}$	f	$V_{pp}$	$V_{avg}$
RP-C7.6-ST with 1 kg	10 V/div	5 s/div	3.33 Hz	40.4 V	−7.67 V
RP-C7.6-ST with 2 kg	10 V/div	5 s/div	3.33 Hz	40.4 V	−7.67 V
RP-C7.6-ST with 3 kg	10 V/div	5 s/div	185 mHz	5.65 V	−4.84 V
RP-C7-LT with 1 kg	10 V/div	5 s/div	145 mHz	11.9 V	−5.85 V
RP-C7-LT with 2 kg	10 V/div	5 s/div	145 mHz	11.9 V	−5.85 V
RP-C7-LT with 3 kg	10 V/div	5 s/div	145 mHz	11.9 V	−5.85 V
FSR400 with 1 kg	10 V/div	5 s/div	285 mHz	11.1 V	−2.22 V
FSR400 with 2 kg	10 V/div	5 s/div	285 mHz	11.1 V	−2.22 V
FSR400 with 3 kg	10 V/div	5 s/div	291 mHz	8.28 V	−4.44 V

**Table 12:** Bending-moment sensor.

	Flex4.5
	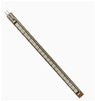
Dimensions	112 mm × 6.35 mm × 0.43 mm
Operating voltage	3.3–5 V DC
Life cycle	>1 million
Flat resistance	10 K Ohms ± 30%
Bend resistance	60 K–110 K Ohms ± 30% (@ 180° pinch bend)
Power rating	0.5 Watts continuous; 1 Watt Peak
Temperature	−35 °C~+80 °C

**Table 13:** Waveform results for sensors underwent finger movements.

	Test Action	Oscilloscope Signal
Slight bending	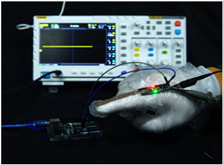	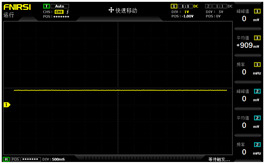
Mild bending	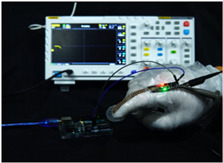	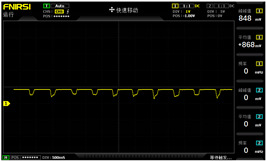
Moderate bending	** 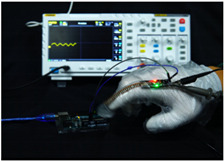 **	** 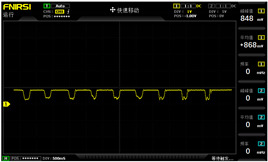 **
Severe bending	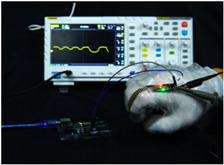	** 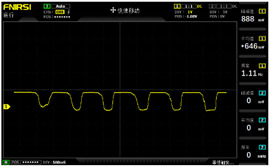 **

**Table 14:** Heart-rate pulse sensors.

	Pulse Sensor	Intelligent Watch
	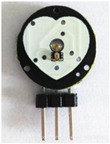	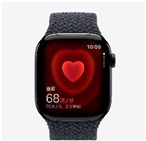
Size	16 mm	42 mm
Power supply	3.3~5 V	3.3~5 V
Current consumption	~4 ma	~300 ma
Output signal type	Analog signal	Table data
Signal type detected	Photoplethysmography (PPG)	Optical heart rate monitoring and electrocardiogram (ECG) monitoring

## Data Availability

Data unavailable due to privacy restrictions.
